# The assessment of left ventricular volume and function in gated small animal ^18^F-FDG PET/CT imaging: a comparative study of three commercially available software tools

**DOI:** 10.1186/s13550-023-01026-w

**Published:** 2023-08-12

**Authors:** Mathias J. Zacherl, Agus Simenhandra, Magdalena Lindner, Peter Bartenstein, Andrei Todica, Guido Boening, Maximilian Fischer

**Affiliations:** 1 Department of Nuclear Medicine, Ludwig-Maximilians-University, 81377 Munich, Germany; 2 Department of Cardiology, Medical Clinic and Polyclinic I, University Hospital Munich, Marchioninistraße 15, 81377 Munich, Germany; 3 DZHK (German Centre for Cardiovascular Research), Partner Site Munich Heart Alliance, 80336802 Munich, Germany

**Keywords:** Small-animal positron emission tomography, ^18^F-FDG, Heart function, Imaging software, QGS, MIM, PMOD

## Abstract

**Background:**

Several software tools have been developed for gated PET imaging that use distinct algorithms to analyze tracer uptake, myocardial perfusion, and left ventricle volumes and function. Studies suggest that different software tools cannot be used interchangeably in humans. In this study, we sought to compare the left ventricular parameters in gated ^18^F-FDG PET/CT imaging in mice by three commercially available software tools: PMOD, MIM, and QGS.

**Methods and results:**

Healthy mice underwent ECG-gated ^18^F-FDG imaging using a small-animal nanoPET/CT (Mediso) under isoflurane narcosis. Reconstructed gates PET images were subsequently analyzed in three different software tools, and cardiac volume and function (end-diastolic (EDV), end-systolic volumes (ESV), stroke volume (SV), and ejection fraction (EF)) were evaluated. While cardiac volumes correlated well between PMOD, MIM, and QGS, the left ventricular parameters and cardiac function differed in agreement using Bland–Altman analysis. EDV in PMOD vs. QGS: *r* = 0.85; *p* < 0.001, MIM vs. QGS: *r* = 0.92; *p* < 0.001, and MIM vs. PMOD: *r* = 0.88; *p* < 0.001, showed good correlations. Correlation was also found in ESV: PMOD vs. QGS: *r* = 0.48; *p* = 0.07, MIM vs QGS: *r* = 0.79; *p* < 0.001, and MIM vs. PMOD: *r* = 0.69; *p* < 0.01. SV showed good correlations in: PMOD vs. QGS: *r* = 0.73; *p* < 0.01, MIM vs. QGS: *r* = 0.86; *p* < 0.001, and MIM vs. PMOD: *r* = 0.92; *p* < 0.001. However, EF among correlated poorly: PMOD vs. QGS: *r* = −0.31; *p* = 0.26, MIM vs. QGS: *r* = 0.48; *p* = 0.07, and MIM vs. PMOD: *r* = 0.23; *p* = 0.41. Inter-class and intra-class correlation coefficient were > 0.9 underlining repeatability in using PMOD, MIM, and QGS for cardiac volume and function assessment.

**Conclusions:**

All three commercially available software tools are feasible in small animal cardiac volume assessment in gated ^18^F-FDG PET/CT imaging. However, due to software-related differences in agreement analysis for cardiac volumes and function, PMOD, MIM, and QGS cannot be used interchangeably in murine research.

**Supplementary Information:**

The online version contains supplementary material available at 10.1186/s13550-023-01026-w.

## Background

Many software tools have been developed and are essential for the clinical and preclinical analysis of single photon emission computed tomography (SPECT) and positron emission tomography (PET) imaging.

The commercially available software covers various research areas, including oncology [1–3], neurology [4, 5], and cardiovascular entities [6–9]. Software tools bear great potential to analyze data by automated anatomical volume of interest quantifications [10].

However, comparing software applications is still challenging and can result in different dosimetry analyses of clinical data derived from peptide receptor radionuclide therapy (PRRT) using ^177^Lu-DOTATATE [11]. For over a decade, tools for automated quantifying myocardial ischemia and wall motion defects in cardiac SPECT imaging have offered valid programs and algorithms [12]. However, differences in certain features and artifacts might need to be manually corrected by the user despite automated processing [13]. ^82^Rubidium PET myocardial perfusion quantification in patients was recently used to validate the novel Carimas software [14]. Cross-comparison studies of compartment models in hypertrophic cardiomyopathy (HCM) used the software tools Carimas, Flowquant, and PMOD to evaluate ^13^N-ammonia PET for myocardial perfusion. This study showed consistent global and regional myocardial blood flow (MBF) values. However, there was significant variability in segmental values supplied by the circumflex branch of the left coronary artery. These differences limit the interchangeability of the studied software tools [15]. Another study quantified the MBF in HCM patients. It showed that PMOD and QPET (Cedars Sinai) could not be used interchangeably due to anatomic characteristics in HCM patients compared to non-HCM patients [16].

Comparison of QPET, syngo MBF, and PMOD resulted in excellent correlations in myocardial flow reserve (MFR) in ^13^N-ammonia PET and the respective vascular territories [17]. However, reproducibility for other cardiac tracers is still challenging. In previous reports, ^82^rubidium imaging quantification depended on software tools (e.g., PMOD, FlowQuant, and syngo MBF) [18].

Gated data acquisition in myocardial perfusion SPECT and PET allows analysis of wall motion and calculation of left ventricular volumes, including end-diastolic (EDV), end-systolic volumes (ESV), and left ventricular ejection fraction (EF). Left ventricular cardiac volumes and EF are reliable prognostic parameters in patients [19]. In the clinical setting, ^18^F-FDG represents a widely used tracer detecting hibernating myocardium [20, 21], prosthetic valve endocarditis and device infections [22, 23], and sarcoidosis [24, 25]. Previous clinical studies comparing QGS and 4D-MSPECT to magnetic resonance imaging (MRI) showed a good agreement for EDV, ESV, and EF in patients with coronary heart disease [26, 27]. Several validated software packages are commercially available for humans. In addition, the ^18^F-FDG tracer can also be utilized in small animal PET research for the detection of myocardial defects and to assess murine heart function [28–30].

To date, no studies are comparing different software tools for cardiac volumes and function in basic murine research.

In this study, we sought to compare the left ventricular parameters using PMOD, MIM, and QGS in cardiac small animal ^18^F-FDG PET/CT imaging, thereby contributing to the existing literature on its feasibility and differences and assessing interchangeability.

## Material and methods

### Mice

Male C57/Bl6J mice were purchased from Charles River Laboratories (Sulzfeld, Germany). In total, 15 healthy mice, 8 male and 7 female, at 8 weeks of age were used. Mice's heart rate was 492 ± 53 beats per minute.

Animal care and all experimental procedures were performed according to the Guideline for the Care and Use of Laboratory Animals published by the US National Institutes of Health (NIH Publication No. 85-23, revised 1996). Study protocols complied with the institution's guidelines and were approved by the Government's animal ethics committee.

### PET/CT imaging and reconstruction

ECG-gated ^18^F-FDG-PET/CT scans were performed using a small-animal PET/CT scanner (nanoPET/CT; Mediso). The animals had free access to food and water until before the scan, as described previously [30–32]. No prior fasting was used in the protocol due to enhanced 18F-FDG uptake upon isoflurane anesthesia and exclusion of further evaluation regarding tracer uptake such as SUVs/cardiac injected activity per gram. Bedding was changed regularly to avoid ingestion of bedding.

Anesthesia was induced (2.5%) and maintained (2.0%) with isoflurane delivered in pure oxygen at a rate of 1.5 L/min via a face mask. The core body temperature was maintained within the normal range using a heating pad and monitored by a rectal thermometer.

After placing an intravenous catheter into a tail vein, approximately 20 MBq of ^18^F-FDG was injected in a volume of ~ 0.1 ml. The catheter was then flushed with 0.05 ml of isotonic saline solution. Animals remained anesthetized during the entire scan and were placed in a prone position within the PET/CT scanner.

Modified neonatal needle ECG electrodes (Kendall, Cardinal Health, Dublin, Ireland) were placed into both forepaws and the left hind paw. An integrated physiological monitoring system recorded the ECG signal and vital parameters.

First, a CT scan (semicircular full trajectory, maximum field of view, 480 projections, scan mode helical, pitch 1.0, X-ray power 35 kVp × 900 µA, exposure per projection 170 ms, and 1:4 binning) was acquired for attenuation correction. The ECG-gated PET recording was initiated 30 min after the tracer injection and lasted 15 min. Recovery from anesthesia and the PET/CT scan was monitored closely by a veterinarian. Gated mouse PET studies were reconstructed using the Tera-Tomo 3-dimensional reconstruction algorithm (Nucline NanoScan, Mediso), which includes point-spread correction and the following settings: 8 iterations, 6 subsets, normal regularization, median and spike filter on, edge artifact reduction on, voxel size of 0.5 mm, and 400- to 600- keV energy window, and coincidence mode 1–3. Gating parameters were set at 16 frames. All PET data were corrected for randoms, scatter, attenuation, and decay.

### PET image processing and analysis

#### QGS workflow

Gated mouse PET images were processed as described previously [30]. Analysis of the gated PET images in QGS® (Cedars-Sinai, Los Angeles, CA, USA) required an image adjustment by a scaling factor to approximate human dimensions and contour detection by the automated software [33]. We used a similar approach by a Python script adjusting the voxel size in the x,y, and z-axis by tenfold in DICOM files. Left ventricular function parameters: end-diastolic (EDV), end-systolic (ESV) the stroke volume (SV), and the left ventricular ejection fraction (EF), were calculated from ECG-gated images using QGS® software, as described previously [33].

#### PMOD workflow

Reconstructed whole body PET files were imported into PMOD (Version 3.8, Ltd., Zurich, Switzerland). An automated protocol for mice was used for gated image analysis. PMOD automatically converts the ECG-gated files into dynamic PET images. Endocardial and epicardial contours were automatically traced in the PET images. Based on the contours, various parameters of the left ventricle were calculated: EDV, ESV, SV, and EF.

#### MIM workflow

MIM (Version 7.1.11) was used for image registration and atlas-based segmentation to generate left ventricular myocardial contours. Gated whole-body PET images were adjusted by a scaling factor using the Python script to enable analysis in the automated cardiac PET mouse workflow provided by the company.

After manually adjusting the heart’s orientation (short axis, horizontal long axis, and vertical long axis), an automated registration is performed to match the heart registration and define the contours. Left ventricular parameters (EDV, ESV, SV, and EF) were automatically calculated.

### Statistical analysis

All results are reported as mean ± standard deviation (SD). Statistical analysis was performed with Prism (Version 9, GraphPad Software, LLC) and IBM SPSS Statistics (Version 29.0.0.0 (241)). No outlier correction was performed in the dataset. The Wilcoxon signed rank, or the Mann–Whitney *U* test, was applied for groups without normal distribution. EDV, ESV, SV, and EF were analyzed using matched one-way ANOVA with Sidaks multiple comparison test. The correlation was calculated using Pearson correlation coefficients between two datasets; scatter diagrams and Bland–Altman plots showing difference vs. average were used for visualization and further analysis of bias and agreement. To assess the intra- and inter-reader reproducibility, cardiac volume assessment in all three software applications were repeated 10 weeks after the initial review by the same reviewer and by a second reviewer, for the calculation of intraclass correlations coefficients (ICCs) with 95% confidence intervals (CI). The studies were presented in a random order. Differences were considered statistically significant at a p-value of 0.05.

## Results

### Cardiac volume and function in QGS vs PMOD vs MIM

**Fig. 1:**
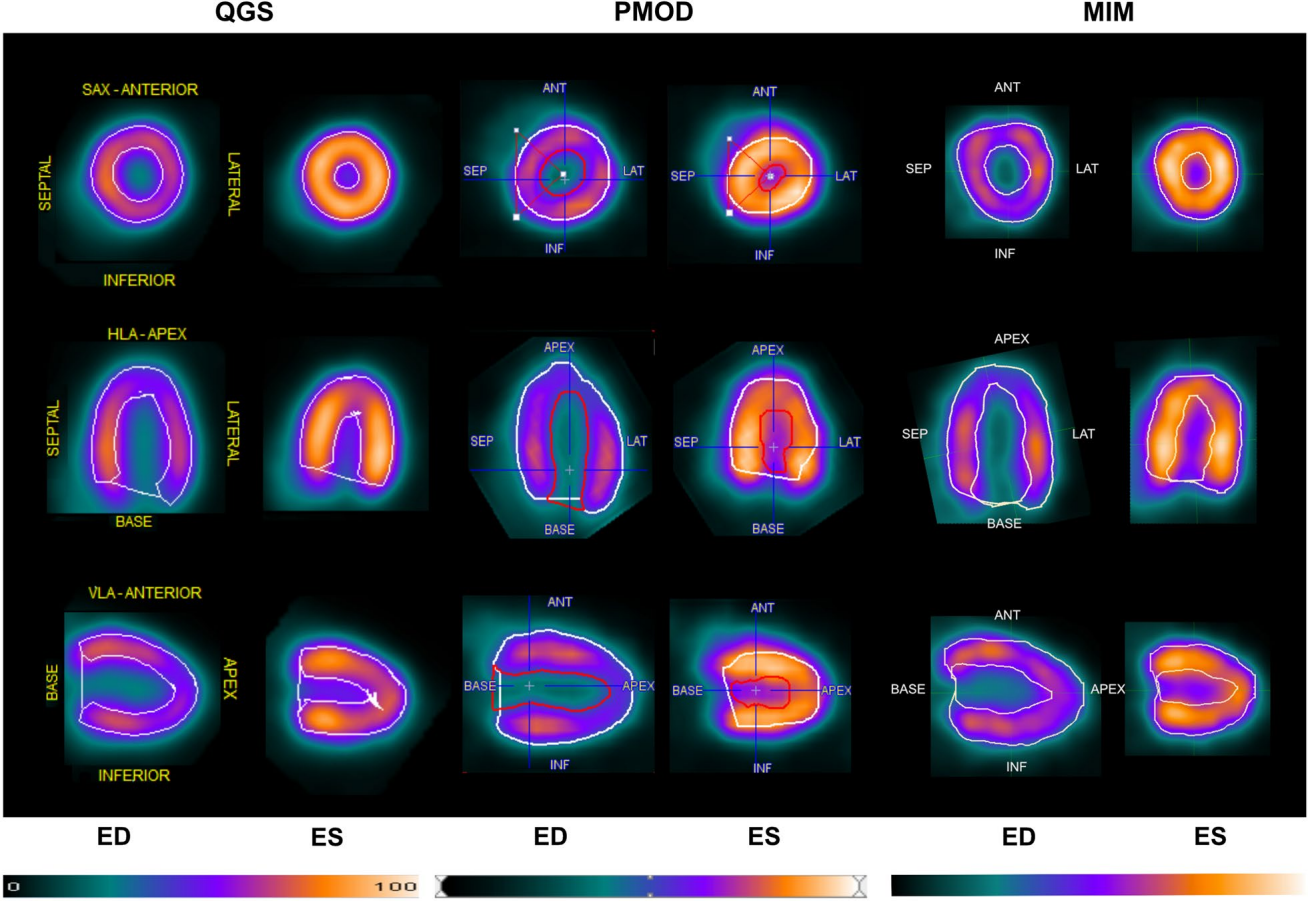
Cardiac small animal ^18^F-FDG PET images in QGS, PMOD, and MIM. Representative cardiac PET images illustrating the commercially available software QGS (left), PMOD (middle), and MIM (right) in the same healthy mouse, each in end-diastole (ED) and end-systole (ES). The upper row shows short axis view (SAX). The middle row shows the horizontal long axis (HLA), and the bottom row illustrates the  vertical long axis (VLA). Anterior (ANT), septal (SEP), lateral (LAT), inferior (INF). Color scale ranging from 0 to 100% for each software are displayed below

**Fig. 2:**
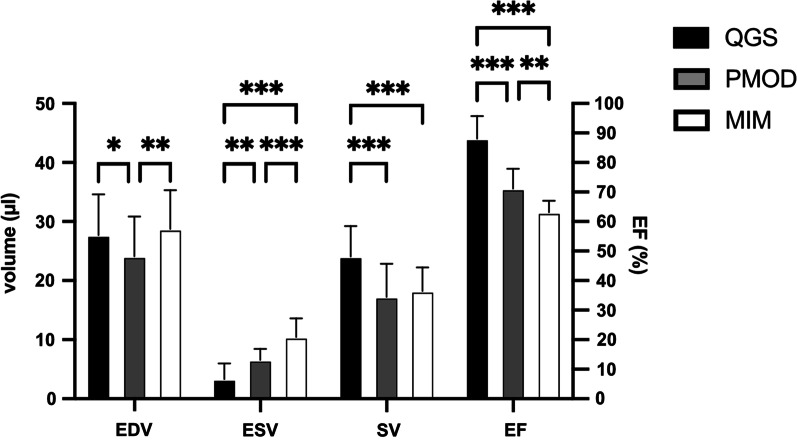
Assessment of cardiac volumes and function illustrates differences between software tools. Comparison of cardiac PET parameters (EDV, ESV, SV, and EF) in QGS (in black), PMOD (in grey), and MIM (in white). The left y-axis depicts volume assessment; the right y-axis corresponds to EF. Each group consists of *N* = 15. Data represents mean ± SD. *p < 0.05, **p < 0.01, ***p < 0.001

Gated mouse ^18^F-FDG PET images could be analyzed using all three commercially available software packages. Figure 1 illustrates representative PET images after the DICOM file importing into the respective software tools. Each application could identify the cardiac cycle and determine end-diastole and end-systole. Next, we assessed statistically relevant differences among the values generated from the same gated datasets when imported and analyzed in QGS, PMOD, or MIM. The datasets comprised 8 male and 7 female mice at 8 weeks. Interestingly, several differences comparing our dataset were evident regarding EDV, ESV, SV, and EF. These results are displayed in Fig. 2. The EDV between QGS vs. PMOD (*p* < 0.05) and PMOD vs. MIM (*p* < 0.01) differed significantly, while QGS vs. MIM showed similar results (*p* = n.s.). ESV was different among all three software applications (*p* < 0.01). Regarding the SV, QGS differed significantly from PMOD (*p* < 0.001) and MIM (*p* < 0.001). SV among PMOD and MIM resulted in similar results (n.s.). Interestingly the EF in QGS was consistently higher compared to PMOD (*p* < 0.001) and compared to MIM (*p* < 0.001). However, PMOD vs. MIM also showed differences in EF (*p* < 0.01). The mean values, including standard deviation and range for EDV, ESV, SV, and EF, are displayed in Table 1. The inter-class correlation coefficient for QGS was 0.99; 95% CI 0.998–0.999, for PMOD 0.973; 95% CI 0.955–0.984, and for MIM 0.998; 95% CI 0.997–0.999, indicating excellent ICC results. The intra-class correlation coefficient for QGS was 0.997; 95% CI 0.996–0.999, for PMOD 0.982; 95% CI 0.970–0.989, and for MIM 0.992; 95% CI 0.982–0.996, furthermore showed good to excellent repeatability in all three applications [34].

**Table 1:** EDV, ESV, SV, and EF values from gated mouse 18F-FDG PET/CT images

Parameter	QGS		PMOD		MIM	
	Mean ± SD	Range	Mean ± SD	Range	Mean ± SD	Range
EDV (µl)	28 ± 6.9	21–43	24 ± 6.7	17–35	29 ± 6.6	22–43
ESV (µl)	3.5 ± 2.7	0–9	6.7 ± 1.9	4.2–10	11 ± 3.2	8–18
SV (µl)	24 ± 5.2	19–34	17 ± 5.6	10–28	18 ± 4.0	14–26
EF (%)	88 ± 8.7	74–98	71 ± 6.8	60–81	63 ± 3.9	55–69

The range is defined as a minimum to maximum

Regarding gender differences, we detected slightly bigger values in EDV in male vs. female mice using each software application (QGS, PMOD, and MIM; *p* < 0.05). Other cardiac volumes such as ESV, SV, and EF did not differ (Additional file 1: Figure S1). Since we cumulated the cardiac volumes and function parameters to be evaluated in the respective software applications, no systematic bias should be evident.

### Correlation, agreement, and bias of gated ^18^F-FDG PET/CT: QGS vs PMOD vs MIM

**Fig. 3:**
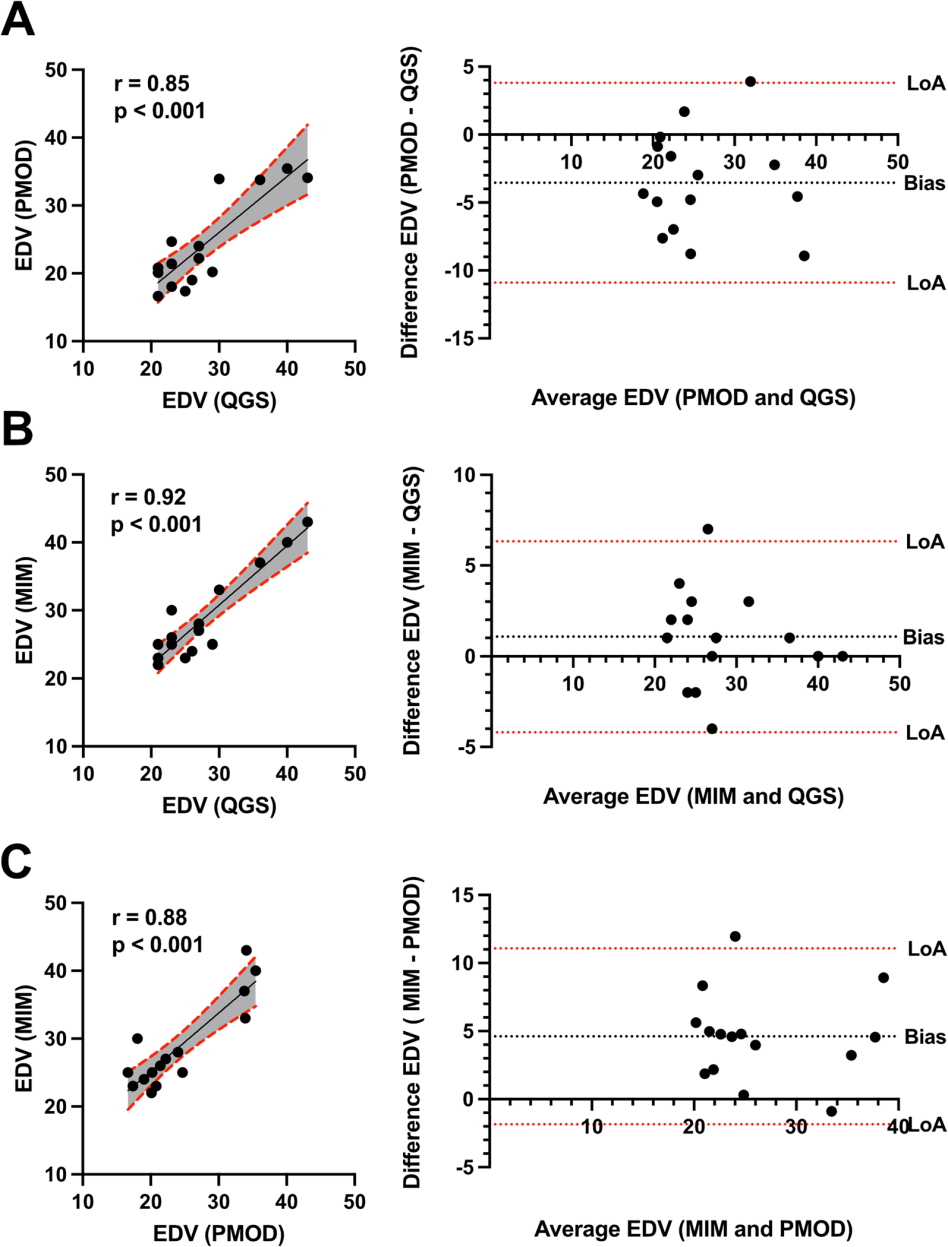
Correlation and Bland–Altman analysis of end-diastolic volume (EDV) showing bias and agreement. Scatter plots illustrate the correlation of EDV among QGS, PMOD, and MIM. **A** PMOD vs QGS, **B** MIM vs QGS, and **C** MIM vs PMOD. A 95% confidence interval is plotted in grey color. Corresponding Bland–Altman-plot on the right side. Dotted lines display the limits of agreement (LoA; ± 1.96 SD) and bias

Correlation and Bland–Altman analysis of cardiac volume and function parameters were evaluated across the software tools to analyze the numeric differences further. The correlation analysis of EDV between QGS and PMOD results in good correlation (*r* = 0.85, *p* < 0.001, Fig. 3A) and -3.55 bias. MIM and QGS showed an even better correlation (*r* = 0.92, *p* < 0.001, Fig. 3B) and 1.07 bias. At the same time, PMOD and MIM also showed a decent correlation (*r* = 0.88, *p* < 0.001, Fig. 3C) and 4.61 bias.

**Fig. 4:**
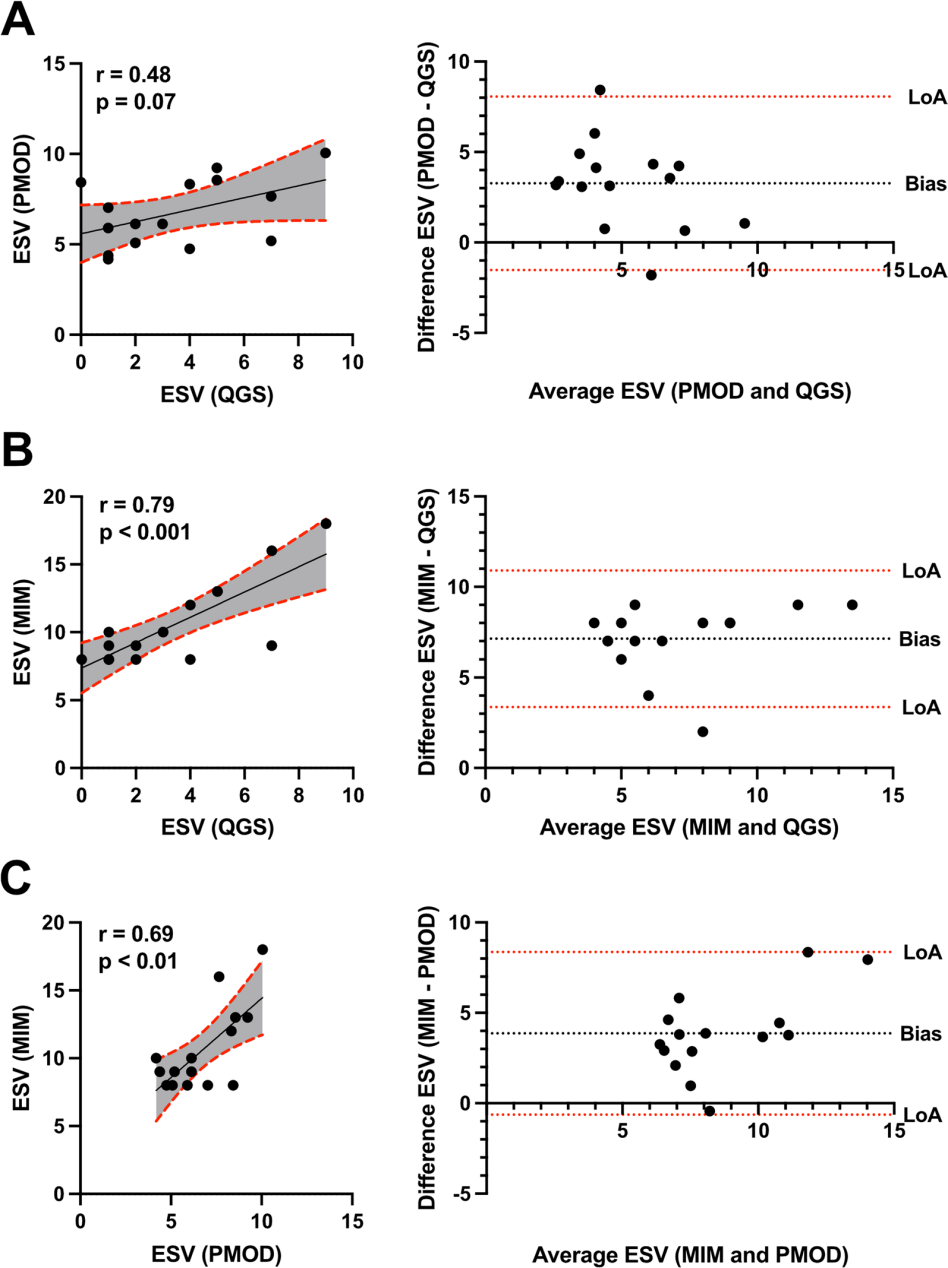
Correlation and Bland–Altman analysis of end-systolic volume (ESV) showing bias and agreement. Scatter plots illustrate the correlation of ESV among QGS, PMOD, and MIM. **A** PMOD vs QGS, **B** MIM vs QGS, and **C** MIM vs PMOD. A 95% confidence interval is plotted in grey color. Corresponding Bland–Altman-plot on the right side. Dotted lines display the limits of agreement (LoA; ± 1.96 SD) and bias

Next, the ESV showed a tendency for correlation in PMOD compared to QGS (*r* = 0.48, *p* = 0.07, Fig. 4A) and 3.2 bias. In contrast, MIM vs. QGS displayed a good correlation (*r* = 0.79, *p* < 0.001, Fig. 4B) and 7.1 bias. MIM vs. PMOD showed a moderate correlation in (*r* = 0.69, *p* < 0.01, Fig. 4C) and 3.8 bias.

**Fig. 5:**
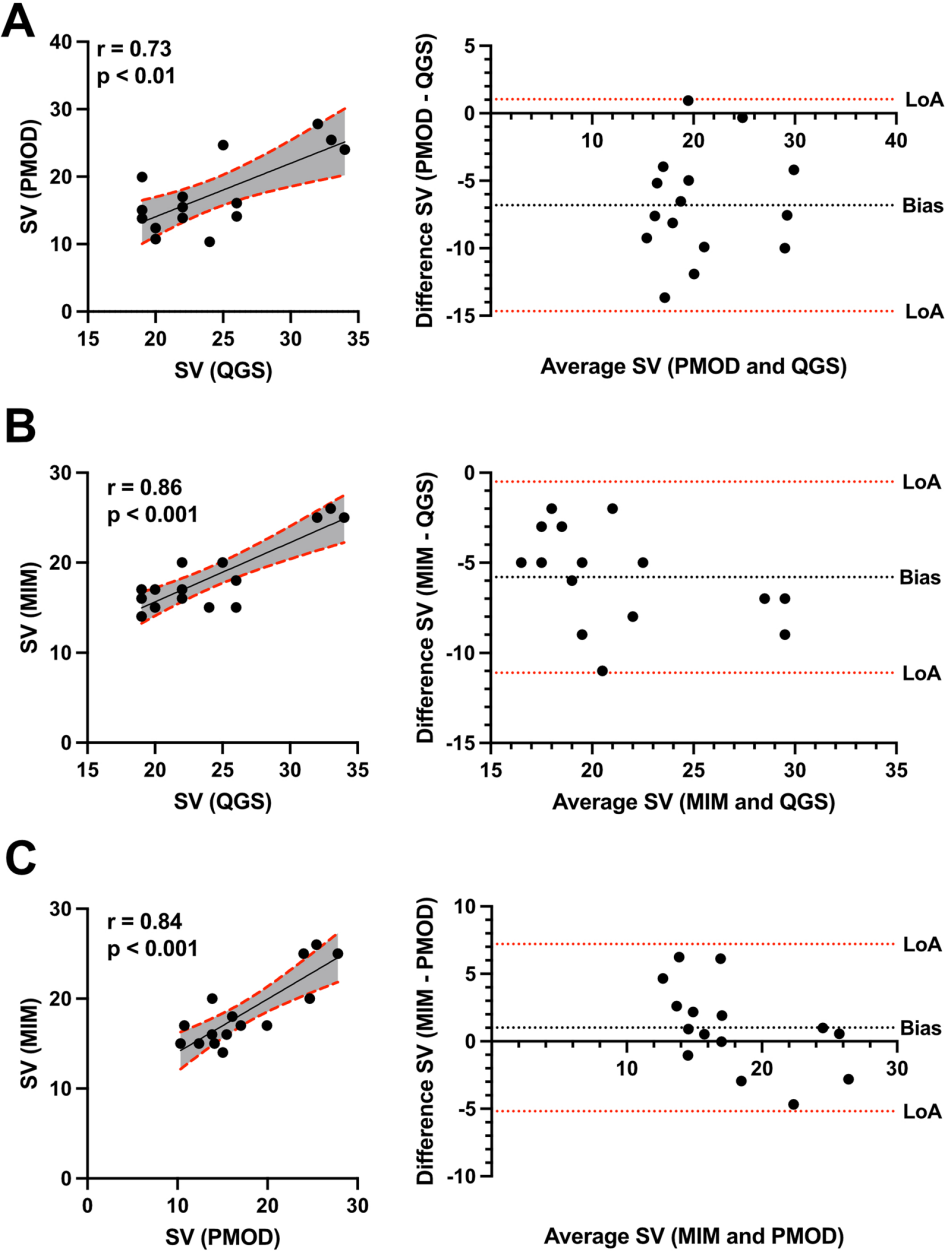
Correlation and Bland–Altman analysis of stroke volume (SV) showing bias and agreement. Scatter plots illustrate the correlation of SV among QGS, PMOD, and MIM. **A** PMOD vs QGS, **B** MIM vs QGS, and **C** MIM vs PMOD. A 95% confidence interval is plotted in grey color. Corresponding Bland–Altman-plot on the right side. Dotted lines display the limits of agreement (LoA; ± 1.96 SD) and bias

Comparing SV between the PMOD and QGS software showed a good correlation (*r* = 0.73, *p* < 0.01, Fig. 5A) and -6.8 bias. While MIM vs. QGS resulted in a good correlation (*r* = 0.86, *p* < 0.001, Fig. 5B) and -5.8 bias, MIM vs. PMOD showed a good correlation (*r* = 0.84, *p* < 0.001, Fig. 5C) and 1.0 bias.

**Fig. 6:**
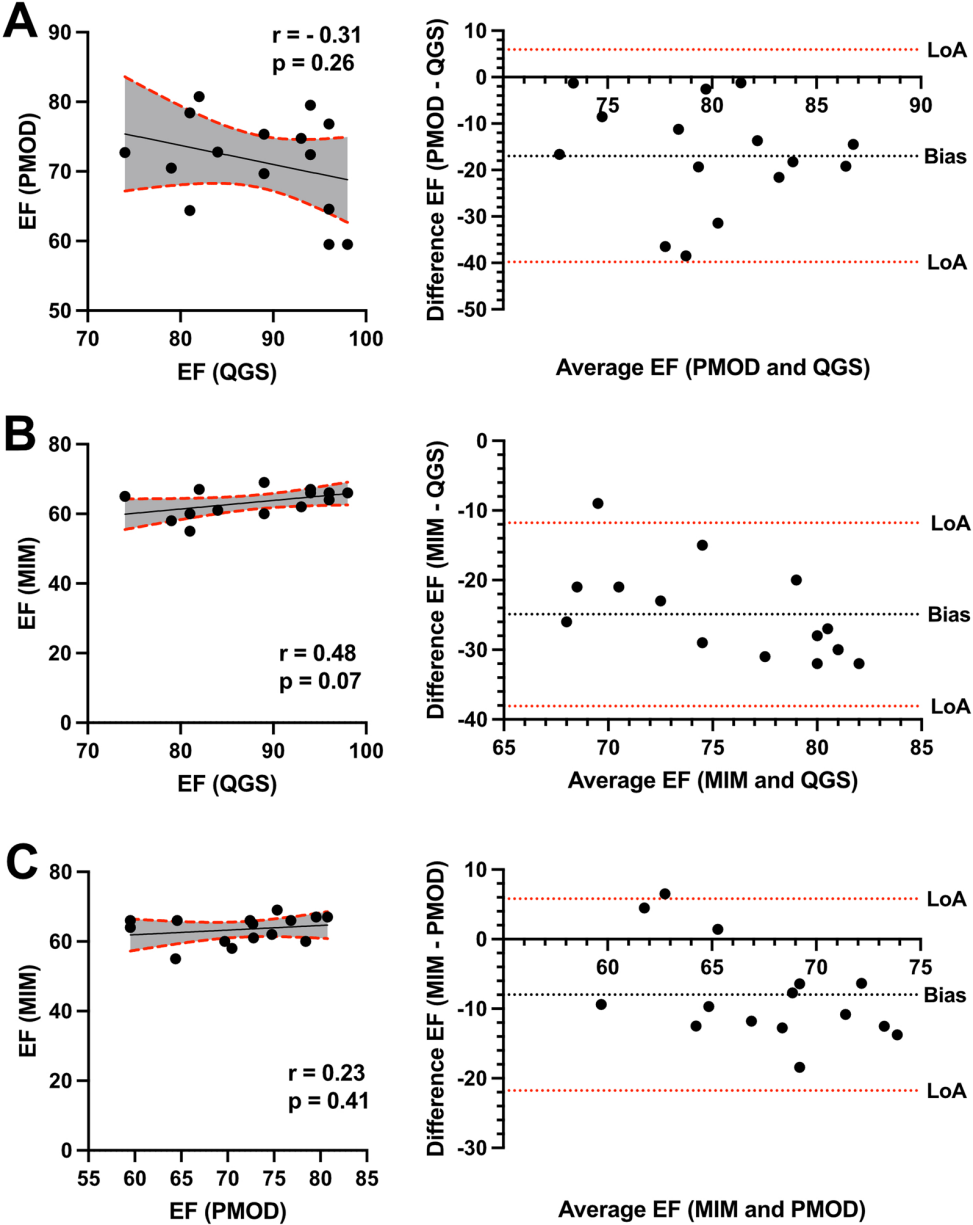
Correlation and Bland–Altman analysis of ejection fraction (EF) showing bias and agreement. Scatter plots illustrate the correlation of EF among QGS, PMOD, and MIM. **A** PMOD vs QGS, **B** MIM vs QGS, and **C** MIM vs PMOD. A 95% confidence interval is plotted in grey color. Corresponding Bland–Altman-plot on the right side. Dotted lines display the limits of agreement (LoA; ± 1.96 SD) and bias

Finally, the cardiac function depicted by EF was correlated. PMOD vs. QGS showed no correlation in (*r* = −0.31, *p* = 0.26, Fig. 6A) and −17.0 bias. Similar results were evident in MIM vs. QGS (*r* = 0.47, *p* < 0.07, Fig. 6B) and −24.9 bias and in MIM vs. PMOD (*r* = 0.23, *p* = 0.41, Fig. 6C) and −7.9 bias.

## Discussion

In this study, we sought to evaluate three commercially available software applications frequently used in patients' cardiac research for volume and function assessment. Our work is the first to compare software tools PMOD, MIM, and QGS in a small-animal study. We demonstrate that using each program in the cardiac ^18^F-FDG gated PET imaging setting is feasible.

We used isoflurane narcosis enhancing ^18^F-FDG uptake in mice hearts to facilitate good imaging quality of the left ventricle [30, 35, 36]. Our ^18^F-FDG-derived cardiac volumes match previous literature using ^13^N-ammonia PET and ^99m^Tc-sestamibi SPECT in mice [7]. We detected excellent correlation for EDV, and Bland Altman analysis showed decent bias (< 5). This software comparability was also evident in ESV among MIM vs. QGS and MIM vs. PMOD. But not to a reasonable extent in PMOD vs. QGS. At the same time, the analysis of SV in all three applications showed good correlations. However, we could show a systematic bias comparing the software tools in Bland Altman's analysis. QGS, compared to PMOD and MIM, showed a systematic overestimation of SV indicated by Bland Altman's analysis of differences vs. averages.

Especially, QGS shows high values for EF compared to PMOD and MIM. Interestingly, no correlation and high bias were recorded in comparing the EF. Therefore, in addition to the systematic bias in SV, this argues for different anatomical discrimination of the left ventricle due to the partial volume effect. While PMOD enables the selection of distinct species, such as humans, rats, mice, and pigs, the MIM and QGS software is designed for humans only. The software tools used in this study provide fully automated slice orientations, ventricular segmentation, endo- and epicardial contouring, and polar map generation compared to established databases. However, since MIM and QGS were developed for human hearts, that could present technical limitations in the technological transfer to mouse hearts. The volumes in our eight weeks old mice in end-systole refer to approximately 10 µl or less, which might challenge the resolution and accuracy of gated small-animal PET images. Moreover, we showed that more significant volumes, such as the EDV, validly correlate and show good agreement in the Bland–Altman analysis. Interestingly, MIM seems to underestimate EF in mice systematically. MIM is a relatively new software application and limited literature is available for cardiac research. This study is the first to describe its potential utility in preclinical murine cardiac research. Since MIM was created for human research and even in this setting no MRI reference was publicly available. Our approach to evaluate mice hearts with MIM is novel, challenging and needs further studies with rigorous peer-review. The potential side-effect using a human atlas-based software in evaluating mice hearts can’t technologically solved by our first study. However, the aim of this study was also to examine MIM compared to PMOD, an established research platform showing comparable cardiac volumes.

We used a python script for rescaling for the assessment of mouse cardiac volumes in QGS and MIM. Previously, Croteau et al. showed that excellent agreement between endocardial volumes determined by ^18^F-FDG PET in rats to the actual volume of the cardiac phantom [33]. Croteau et al. showed good agreement between PET and echocardiography for left ventricular volume and left ventricular ejection fraction. Therefore, we assume that the rescaling process used in this study should not interfere with the accuracy of the reconstructed images. However, this is also underlining the limitation of validating our data using other imaging technique.

Previous publications of human data showed excellent correlation and agreement between gated ^18^F-FDG PET and MRI regarding EDV and ESV using either algorithm [26]. Interestingly EF estimated by QGS correlated better with MRI, while 4D-MSPECT showed no significant underestimation to MRI.

While QGS and PMOD have been commercially available for several years and are well-established, MIM software could be a suitable alternative in cardiac research. At the time of this study, no literature was published that evaluated or used MIM software for cardiac analysis so far. MIM has been used to quantify ^68^ Ga-Pentixafor PET/CT in multiple myeloma [37] and predict treatment response after radioembolization in hepatocellular carcinoma [38]. MIM software has been used in a phase I study of ^68^ Ga-HER2-nanobody for PET/CT of HER2 Expression in breast carcinoma to analyze dosimetry and biodistribution [39] and neurological PET imaging by ^18^F-florbetapir uptake [40]. Previous studies showed that MIM software and PMOD neuro tool in brain amyloid PET imaging by ^18^F-florbetapir can be used interchangeably to calculate standard uptake values [41]*.* Another comparison of both software tools provided comparable quantifications [42]*.*

In addition, quantitative normal MBF and myocardial flow reserve (MFR) from patients were measured by commercially available pharmacokinetic software packages (PMOD, syngo MBF, and FlowQuant) [18]. However, these data consisting of 49 patients showed several statistically significant differences in myocardial perfusion analysis. In patients with hypertrophic cardiomyopathy analyzed for MBF and MFR, the high spillover fractions precluded the interchangeability of PMOD and QPET [16]. It would be tempting to speculate if other software tools would yield higher sensitivity and specificity in clinical diagnosis, e.g., in infective endocarditis and implantable cardiac electronic devices infection [23]. Comparison of software packages for basic researcher is pivotal regarding the amount of resources spent by researcher in both the clinical and pre-clinical setting. A complete, detailed and meticulously description of all features, however, is beyond the scope of this discussion. Despite that, we want to share some thoughts on pro-and cons about the softwares. All three software can run on windows and Mac. QGS and PMOD are both established cardiac research application, and currently more expensive than MIM. A further advantage of MIM is the possibility of weekly, monthly, and yearly subscription that are not available for QGS and PMOD. Time for computing favors QGS and MIM, compared to PMOD. Especially MIM offers the opportunity of self-created and managed workflows enabling good throughput, when analysing animals with the same research question and is very user friendly. Currently QGS is also easy to use and offers all assessment and parameters used in the human cardiac function assessment including shape indexes and left ventricular mechanical dyssynchrony. The PMOD cardiac PET modelling tool offers a tremendous number of parameters including kinetic models, species specific settings (e.g., human, rat, mouse, and pig), that QGS and MIM do not provide. Therefore, at least in our opinion, the most potent research application is currently displayed by PMOD, despite high throughput is time consuming.

The reader should be aware of several limitations in this study. First, our data lack a non-PET imaging external reference control such as magnetic resonance imaging or echocardiography. Second, the number of comparable studies in small animals is minimal, underlining the need for further investigation. Third, potential errors and artifacts in automated processing cannot be excluded since QGS and MIM were programmed for humans, not small animals. That could attribute to high spillover fractions in imaging small mice hearts. Although this study analyzed different software tools, the data was obtained with one scanner and one imaging and reconstruction protocol. Forth, only intravenous FDG application into a tail vein was performed. Intraperitoneal injection was not performed but could represent benefit by avoiding prolongation of isoflurane anesthesia, which may mask efficacy of glucose suppression.

## Conclusions

Analyzing heart volume and function in small animal research is a cornerstone in preclinical cardiac research. Regarding the previous lack of published literature, the present work compares three commercially available software tools for cardiac function in healthy mice using gated ^18^F-FDG PET/CT. Our results indicate that these software tools are feasible in small animal research. The calculated cardiac values are consistent with the published literature. There was a good to excellent correlation for EDV, ESV, and SV comparing the software tools. Bland–Altman analysis showed differences in agreement, limiting the interchangeability of PMOD, MIM, and QGS for murine cardiac volume and function assessment.

### Supplementary Information

**Additional file 1:** Comparison of cardiac PET parameters by gender: male vs. female for EDV (**A**), ESV (**B**), SV (**C**), and EF (**D**) using QGS (in black), PMOD (in grey), and MIM (in white). The left y-axis depicts volume assessment in µl (A to C); y axis in (D) shows the EF in %. Male mice N=8, female mice N=7. Data represents mean ± SD. *p < 0.05, **p < 0.01, ***p < 0.001.

## Data Availability

The authors confirm that the data supporting the findings of this study are available within the article and/or its additional files.

## Abbreviations

18F-FDG: 2-Deoxy-2-[18F] fluoro-D-glucose
CT: Computer tomography
EDV: End diastolic volume
ESV: End-systolic volume
EF: Ejection fraction
PET: Positron emission tomography
SV: Stroke volume
