# Supplementary material for: The assessment of left ventricular volume and function in gated small animal 18F-FDG PET/CT imaging: a comparative study of three commercially available software tools
Source: EJNMMI Res. 2023 Aug 12;13:75. doi: 10.1186/s13550-023-01026-w (PMC10423195; doi:10.1186/s13550-023-01026-w)
Supplement: Supplementary file 1 — Additional file 1: Comparison of cardiac PET parameters by gender: male vs. female for EDV (A), ESV (B), SV (C), and EF (D) using QGS (in black), PMOD (in grey), and MIM (in white). The left y-axis depicts volume assessment in µl (A to C); y axis in (D) shows the EF in %. Male mice N=8, female mice N=7. Data represents mean ± SD. *p < 0.05, **p < 0.01, ***p < 0.001. [file 13550_2023_1026_MOESM1_ESM.docx]

The assessment of left ventricular volume and function in gated small animal ^18^F-FDG PET/CT imaging: a comparative study of three commercially available software tools

Mathias J. Zacherl ^1^, Agus Simenhandra ^2,3^, Magdalena Lindner ^1^, Peter Bartenstein ^1^, Andrei Todica ^1^, Guido Boening ^1^, Maximilian Fischer ^2,3, †^

^1^ Department of Nuclear Medicine, Ludwig-Maximilians-University, Munich 81377, Germany; [Mathias.Zacherl@med.uni-muenchen.de](mailto:Mathias.Zacherl@med.uni-muenchen.de) (M.Z.), [Magdalena.Lindner@med.uni-muenchen.de](mailto:Magdalena.Lindner@med.uni-muenchen.de) (M.L.), [Peter.Bartenstein@med.uni-muenchen.de](mailto:Peter.Bartenstein@med.uni-muenchen.de) (P.B.); [Andrei.Todica@med.uni-muenchen.de](mailto:Andrei.Todica@med.uni-muenchen.de) (A.T.), [Guido.Boening@med.uni-muenchen.de](mailto:Guido.Boening@med.uni-muenchen.de) (G.B.)

^2^ Medizinische Klinik und Poliklinik I, Klinikum der Universität München, Ludwig-Maximilians-Universität, Marchioninistrasse 15, 81377 Munich, Germany; [Agus.Simenhandra@campus.lmu.de](mailto:Agus.Simenhandra@campus.lmu.de) (A.S.), [Maximilian.Fischer@med.uni-muenchen.de](mailto:Maximilian.Fischer@med.uni-muenchen.de) (M.F.)

^3^ DZHK (German Centre for Cardiovascular Research), partner site Munich Heart Alliance, 80336802 Munich, Germany

**^†^ Corresponding author:**

Dr. med. Maximilian Fischer

University Hospital Munich,

Department of Cardiology, Medical Clinic and Polyclinic I,

Marchioninistraße 15,

81377 Munich, Germany.

[Maximilian.Fischer@med.uni-muenchen.de](mailto:Maximilian.Fischer@med.uni-muenchen.de)

**Supplemental figure S1**

Comparison of cardiac PET parameters by gender: male vs. female for EDV (**A**), ESV (**B**), SV (**C**), and EF (**D**) using QGS (in black), PMOD (in grey), and MIM (in white). The left y-axis depicts volume assessment in µl (A to C); y axis in (D) shows the EF in %. Male mice N=8, female mice N=7. Data represents mean ± SD. * p < 0.05, ** p < 0.01, *** p < 0.001.
